# Nanoarchitectonics of the Effects of Curcumin Carbon Dot-Decorated Chitosan Nanoparticles on Proliferation and Apoptosis-Related Gene Expressions in HepG2 Hepatocellular Carcinoma Cells

**DOI:** 10.1021/acsomega.3c03405

**Published:** 2023-09-04

**Authors:** Hasan Ilhan

**Affiliations:** Department of Chemistry, Faculty of Science, Ordu University, Ordu 52200, Turkey

## Abstract

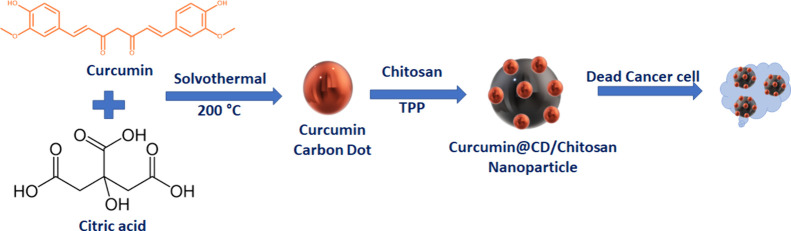
This study examines the potential anticancer properties of curcumin carbon nanodot-decorated chitosan nanoparticles (CCM@CD/CS-NP) in HepG2 hepatocellular carcinoma cells. CCM@CD/CS-NPs were synthesized, and their size, morphology, and elemental analysis were characterized. The combination of curcumin carbon dots and chitosan in the form of a nanoparticle has a number of benefits, including improved solubility and bioavailability of curcumin, enhanced stability and biocompatibility of carbon dots, and sustained release of the drug due to the mucoadhesive properties of chitosan. The purpose of this research was to examine the efficacy of curcumin carbon dot-decorated chitosan nanoparticles as an anticancer agent in the treatment of HepG2 cell lines. The cell proliferation and apoptosis-related gene expressions in HepG2 cells were assessed to investigate the potential use of nanoparticles in vitro. The IC50 value for the inhibitory effect of CCM@CD/CS-NPs on cell growth and proliferation was determined to be 323.61 μg/mL at 24 h and 267.73 μg/mL at 48 h. Increased caspase-3 and -9 activation shows that CCM@CD/CS-NPs promoted apoptosis in HepG2 cells. It was also shown that the overexpression of Bax and the downregulation of Bcl-2 were responsible for the apoptotic impact of CCM@CD/CS-NPs. The nanoparticles have been shown to have minimal toxicity to normal liver cells, indicating their potential as a safe and effective treatment for HepG2. These novel nanomaterials effectively suppressed tumor development and boosted the rate of apoptotic cell death.

## 1 Introduction

Hepatocellular carcinoma (HCC) is one of the leading causes of death from tumoral disorders, which makes it a serious issue for public health around the world.^1^ The cause of this illness can be traced back to a number of different risk factors, some of which are more prevalent in certain regions than others. For example, the spread of hepatitis C virus in the West and Japan is linked to alcoholism, NAFLD, and metabolic syndrome, while the spread of hepatitis B virus in Africa and Asia is largely attributable to aflatoxin B1 consumption^2^

During treatment with radiation or chemotherapy, the most noticeable kind of cell death is apoptosis. The blood vessels, the tongue, the intestines, and the testes are particularly vulnerable to this type of cell death.^3^ Apoptosis is required for homeostasis to occur under normal circumstances. In addition, one of the most important functions of apoptosis is to eliminate precancerous cells and stop the progression of cancer.^4^ The high rate of apoptosis that occurs in normal tissues during chemotherapy and radiation causes severe responses, which might potentially restrict the therapeutic ratio of these modalities.^5^ According to Kostler,^6^ Xerostomia and mucositis develop due to the death of stem cells in the parotid gland and the intestine. Patients with head and neck malignancies or stomach tumors who receive chemotherapy or radiation often have these problems. On the other hand, apoptosis is the most prevalent form of cell death in tumor cells and plays a crucial role in cancer therapy.^7^

Curcumin (CCM) is an extremely effective natural anticancer compound, and it has gained widespread recognition in recent years. Flavoring and preservation are two of its many uses in the food industry.^8,9^ CCM’s beneficial effects on health and biology have made it a household name in the world of alternative medicine for generations.^10−12^ Antioxidant activity (at low doses), protein binding capability, and metal chelating capacity are the three main biological features of curcumin responsible for its therapeutic effects.^13−15^ CCM is extracted from the rhizome of the Zingiberaceae plant Curcuma longa.^16^ The bright yellow appearance of the Curcuma longa rhizome is due to the presence of CCM and other curcuminoids. The use of CCM as a treatment for persistent health issues has shown encouraging results. CCM reduces the symptoms of cancers of the digestive tract, as well as those of the nervous and endocrine systems, diabetes, and cardiovascular disease.^17−22^ Nevertheless, CCM’s poor bioavailability is due to the compound’s insoluble nature in water, its limited absorption via the gastrointestinal tract, and its quick metabolism and elimination.^23,24^

Carbon dots (CDs) are nanomaterials with central carbon atoms that are hybridized between sp2 and sp3 and functional groups surrounding the core. Until now, CD’s structure has been unidentified. The CD is formed from citric acid, ammonium citrate, polyamines, and sugars. The CDs are now used because of their facile production, water dispersibility, minimal cytotoxicity, and great biocompatibility.^25,26^ Size and surface charge make CDs efficient against drug-resistant bacteria, intracellular microorganisms, and biofilms.^27^ CCM-CDs are effective against the swine epidemic diarrhea virus because they inhibit viral entry, negative-strand RNA synthesis, viral budding, and reactive oxygen species (ROS) accumulation. There is some evidence that IFN-stimulating gene proteins and the generation of proinflammatory cytokines are also responsible for the antiviral activity of CCM-CDs.^28^ CCM’s ability to treat metabolic disorders and cardiovascular illnesses has been demonstrated in animal research. CCM is known to modulate a wide variety of biological targets for therapeutic purposes, including transcription factors, protein kinases, inflammatory factors, and enzymes.^29^

Commercially produced chitosan (CS) is a biodegradable cationic polysaccharide with applications in biological medicine, pharmaceuticals, metal materials, and nutritional supplements. The CS manufacturing process uses no harsh chemicals, making it a promising drug delivery route to boost tumor activity.^30^ Due to improved penetration and retention, cancer cells are preferentially targeted by drugs supplied by carbon nanoparticles (CS-NPs).^31^ Jeon and Kim found in vitro and in vivo anticancer efficacy in CS oligomers.^32^ CS-NPs were highly cytotoxic to Calo320, BGC823, BEL7402, and HepG2 colon cancer cell lines.^33,34^ CS-NPs also inhibited sarcoma-180 and hepatoma H22. The data propose a novel HCC medication class.^30^ Nanoparticles for medical applications must meet specific criteria to ensure their effectiveness and safety. Size, shape, surface properties, biocompatibility, and targeting capabilities are key factors. Recent studies^35,36^ emphasize size’s impact on biodistribution, shape’s role in interactions, and surface modifications for stability and targeting. Achieving these requirements enhances the nanoparticle potential for precise diagnostics and therapies. These studies show that successfully synthesized novel chitosan nanoparticles decorated with CCM CDs can activate the CASPASE-3 pathway, decrease BCL-2 expression, and increase BAX expression, all of which contribute to inducing apoptosis in HCC cells. These nanoparticles have also been demonstrated to be relatively nontoxic to healthy liver cells, further supporting their promise as a benign and efficient therapy for HCC.

## 2 Materials and Methods

### 2.1 Materials

The chemicals utilized in the experiment were procured from Sigma-Aldrich and included low molecular weight Chitosan (CS), citric acid, tripolyphosphate pentasodium (TPP), and glacial acetic acid. CCM (*Curcuma Longa* L.) was obtained from a local market in Turkiye. A dialysis tube with a molecular weight cutoff of 2 kDa was procured from Spectra/Por. The additional chemicals utilized in the cell culture and cytotoxicity experiments were specified in the pertinent sections. Deionized water with a conductivity of 18.2 MOhm was utilized in all experimental procedures.

### 2.2 CCM Extract from *Curcuma longa* L. Preparation

Around 50 g of CCM was thoroughly washed under running water, meticulously chopped into small pieces, and then mechanically crushed to a fine powder. In a typical hydrothermal synthesis, the powder of CCM was added to a solution containing 100 mL of water. It was followed by stirring vigorously at 75 °C for 2 h. First, the produced CCM extract was filtered through cotton, and then, it was filtered using Whatman filter paper. These steps were performed in succession. This CCM extract served as the biogenic component in the manufacture of CCM@CDs.

### 2.3 Synthesis of CCM@CDs

In order to create CCM@CDs from CCM extract, a straightforward, one-pot hydrothermal carbonization procedure was performed. In this procedure, the mixture of 15 mL of the CCM extract and 0.2 g of citric acid was taken in an autoclave made of stainless steel, which had a Teflon line with a capacity of 25 mL. The autoclave was heated in a hot air oven at 200 °C for 3 h. When the reaction was finished, the autoclave was allowed to cool down at a temperature of 25 °C. The extract was centrifuged for 1 h at 10,000 rpm after being filtered using Whatman filter paper and allowed to cool to room temperature. The resulting solution, which was dark brown in color and contained CCD@CDs, was collected and then properly maintained in a refrigerator at a temperature of 4 °C for further research purposes.

### 2.4 Synthesis of CCM@CDs/CS-NP

In order to create CS-NP, ionic gelation of CS with TPP anions was used.^33^ The provided method was altered in order to facilitate the scaling-up of nanoparticle production. After overnight stirring at 200 rpm on a magnetic stirrer, the CS was dissolved at a level of 0.4% weight-per-volume (w/v) in acetic acid at a concentration of 1% volume-per-volume (v/v). The mixture was then filtered by using a PVDF syringe filter with pores measuring 0.22 μm. TPP was dissolved in ultrapure water at a level of 1% (w/v), and the solution was then passed through a PVDF membrane syringe filter with pores measuring 0.22 μm. In a pediatric set with a magnetic stirrer set to 700 rpm, the same volume of chitosan and CCM@CDs was cross-linked with TPP in a volume of 1:1. The resulting formulation was put through centrifugation for 10 min at 10,000 rpm, and the pellet was then resuspended in ultrapure water and put through ultrasonication for 100 seconds at 4 degrees Celsius. After centrifugation and ultrasonication were performed a total of three times, the precipitated nanoformulation was lyophilized and kept at a temperature of 4 °C pending further investigation.

### 2.5 Cell Culture

Dulbecco’s Modified Eagle’s Medium (DMEM) with 10% heat-inactivated fetal bovine serum, 20 μg/mL streptomycin, 20 units/mL penicillin, 1 mM sodium pyruvate, and 0.01 mM amino acid solution was used to cultivate human HepG2 hepatocellular carcinoma cells for this work.

The HepG2 hepatocellular carcinoma cells were treated with our novel compound CCM CD-decorated chitosan nanoparticle at a variety of concentrations, including 100, 200, 300, 400, and 500 μg/mL, in order to assess the antiproliferative efficacy at 24 and 48 h according to a time- and dosage-dependent way.

### 2.6 XTT- Cell Proliferation Assay

Our novel compound, CCM CD-decorated chitosan NP, was tested for its antiproliferative effects on HepG2 hepatocellular carcinoma cells at a concentration of 1 × 10^4^ cells in 96-well plates using the XTT (2,3-bis(2-methoxy-4 nitro-5- sulfophenyl)-2H-tetrazolium-5-carboxanilide) assay (Biotium, Cat No: 30007). After the appropriate dosing intervals had passed, the XTT combination was given according to the manufacturer’s instructions. The production of farmazon was evaluated spectrophotometrically at 450 wavelengths (reference wavelength of 630 nm) and colorimetrically using a microplate reader (Multiskan GO microplate spectrophotometer, Termo). As stated by Secme,^37^ absorbance measurements were utilized to determine cell viability (%) using the specified method.
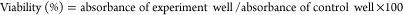


CCM CD-coated chitosan NPs were tested in vitro against HepG2 cells using the IC50 dose–response curve analysis tool at AAT bioquest (https://www.aatbio.com/tools/ic50-calculator).

### 2.7 Real-Time PCR Assay

Isolation of total RNA was carried out on cells from both the control group and the dosage group using Trizol (Hibrigen, Turkey) in line with the instructions provided by the manufacturer. For cDNA synthesis, the Total-Reveal Complete cDNA Synthesis kit was utilized (abm, Cat No: G904, Canada). ABT 2X qPCR SYBER-Green Master Mix (Cat No. Q03–02–05, Turkey) was used in conjunction with RT-PCR (Applied Biosystem, StepOne Plus) to analyze relative mRNA expression levels of apoptosis-related genes, such as BAX, BCL-2, CASPASE-3, and CASPASE-9. As a housekeeping gene, beta-actin was employed to standardize the PCR results. Primers with the same sequences as those found in Kilincarslan Aksoy et al.^38^ and Şirin et al.^39^ were employed, and the sequences of these primers can be found in Table 1.

**Table 1:** Gene Sequences Were Utilized as Primers in This Investigation

gene name	forward	reverse
*Beta-actin*	TCCTCCTGAGCGCAAGTACTC	CTGCTTGCTGATCCACATCTG
*BCL-2*	TGCACCTGACGCCCTTCAC	AGACAGCCAGGAGAAATCAAACAG
*CASPASE-3*	GCAGCAAACCTCAGGGAAAC	TGTCGGCATACTGTTTCAGCA
*CASPASE-9*	GGCTGTCTACGGCACAGATGGA	CTGGCTCGGGGTTACTGCCAG
*BAX*	TCAGGATGCGTCCACCAAGAAG	TGTGTCCACGGCGGCAATCATC

### 2.8 Characterizations

An Agilent/Cary 60 spectrophotometer was used to collect UV–vis absorption spectra. Fourier transform infrared (FT-IR) spectra were collected by using a Varian/660 IR spectrometer. The FEI/TALOS F200S electron microscope was used to capture the HRTEM images at an acceleration voltage of 200 kV. The shape and chemical content of the curcumin carbon dot-modified chitosan nanoparticles were studied by using a Hitachi scanning electron microscope (SU-1510, Hitachi High-Technologies Corp., Tokyo, Japan) in conjunction with energy dispersive X-ray spectroscopy (EDS).

### 2.9 Statistical Analysis

Quantification of the RT-PCR data was accomplished by using the ΔΔ*CT* technique, which was carried out with the assistance of the Gene Globe RT-PCR analysis RT^2^ Profile PCR Array Data Analysis tool (Qiagen). The statistical significance of the comparison between the control group and the dosage group was determined with the use of the Student *t* test, which was based on the results of the RT^2^ Profile PCR Array Data Analysis. All of the findings were summarized using the mean together with the ±standard error, and several additional statistical analyses of the research were carried out.

## 3 Results

### 3.1 Characterization of CCM@CDs/CS-NP

**Figure 1:**
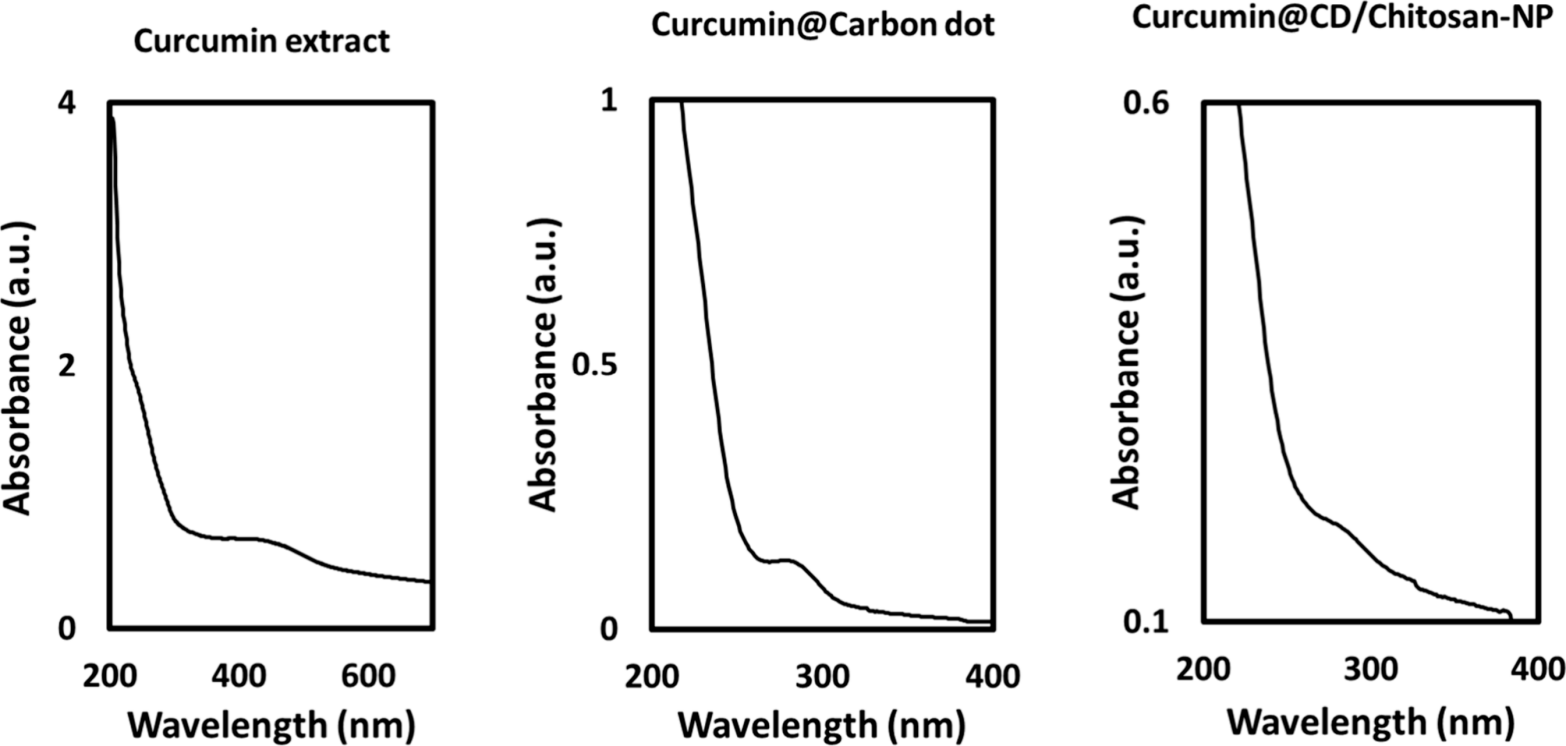
UV–vis spectrum of the CCM extract, CCM@CD, and CCM@CDs/CS-NP.

UV–vis spectroscopy was initially used to evaluate the optical characteristics of CCM@CDs/CS-NP. The particular absorption peaks may be traced directly to the *n*–π* and π–π* transitions. Two absorption bands, at 239 nm (π–π* transition) and 287 nm (*n*–π* transition), are seen in a UV–vis investigation of CCM@CD, demonstrated in Figure 1, whereas two absorption bands, at 260 nm (π–π* transition) and 466 nm (*n*–π* transition), are seen in an evaluation of CCM extract. Because of the surface passivation, the absorbance of the nanoparticles shifted from being at 466 to being at 287 nm. The highest plasmon band at 290 nm is formed by a combination of CCM@CDs and CS-NP.

**Figure 2:**
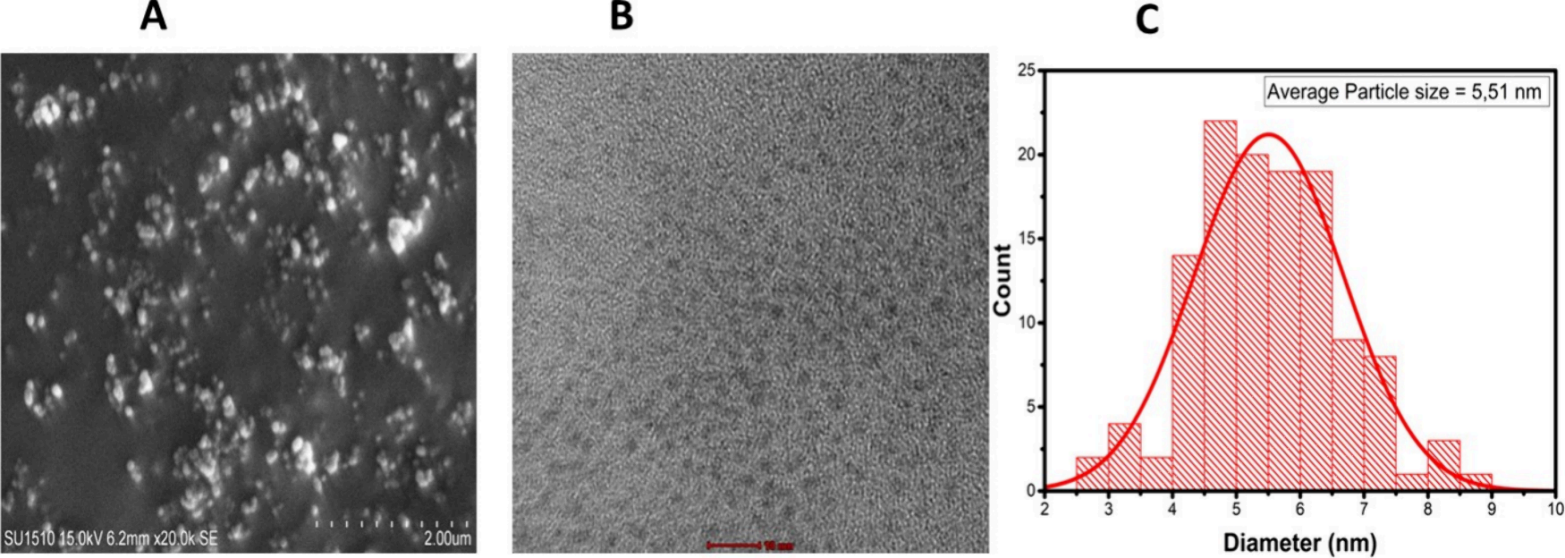
(A) SEM image of CCM@CDs/CS-NP at scale bar 2 μm (B) TEM result of CCM@CDs/CS-NP at scale bar 10 nm, and (C) particle size distribution histogram of CCM@CDs/CS-NP.

High density and a porous shape were seen in the SEM image of CCM@CDs/CS-NP (scale bar, 40 nm) (Figure 2A). High-resolution transmission electron microscopy (HRTEM) pictures (Figure 2B) were acquired at an accelerating voltage of 200 kV by scattering a thin powder of CCM@CDs/CS-NP in ethanol over a carbon-coated copper grid. The surface of CCM CD-coated chitosan nanoparticles has a symmetrical orientation and spherical morphology. A histogram in Figure 2B estimates the average nanoparticles size to be 5,51 nm based on the few powders displayed in the TEM picture.

### 3.2 SEM Elemental Mapping and Composition

**Figure 3:**
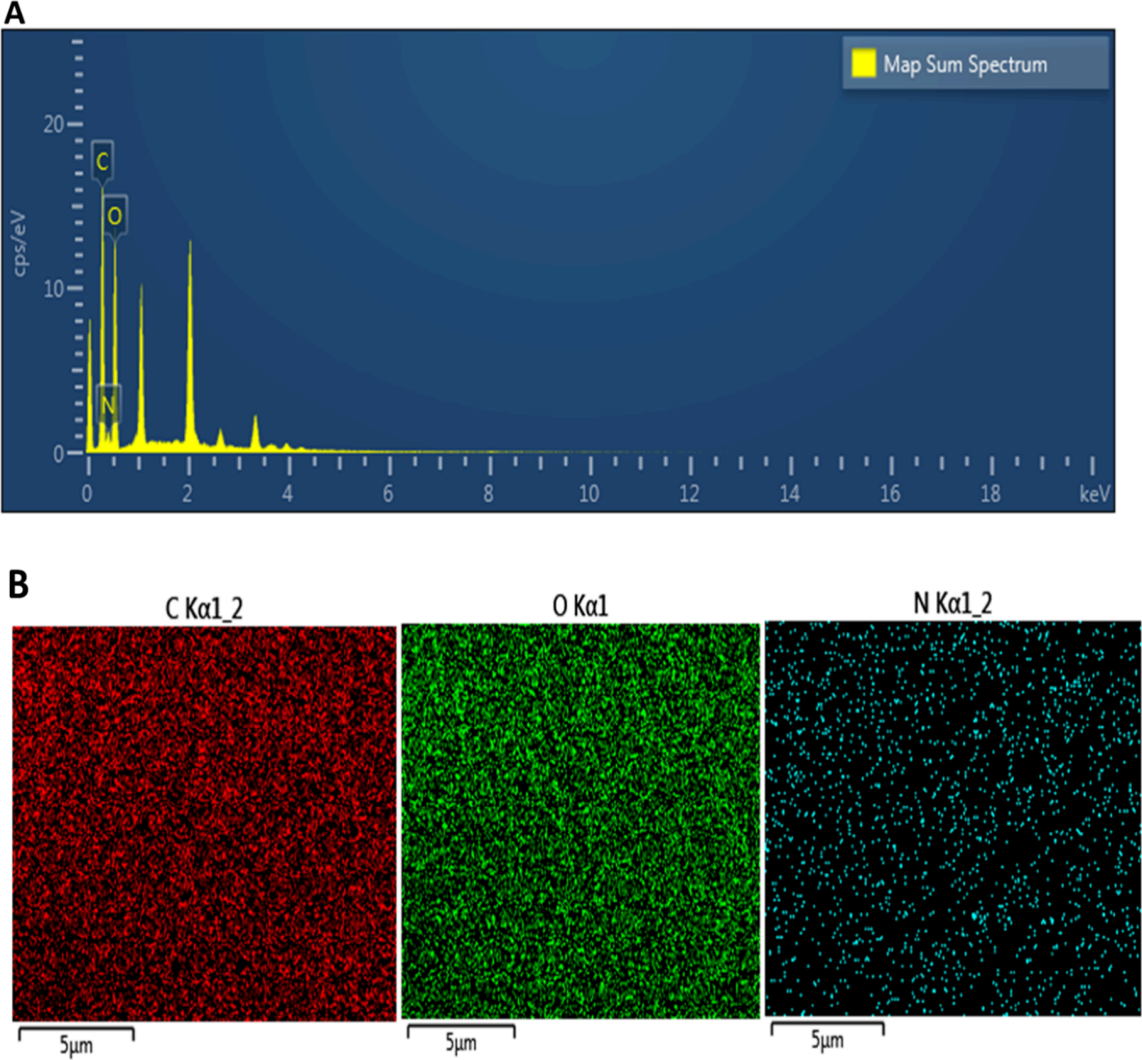
(A) EDX spectrum for CCM@CDs/CS-NP and (B) SEM mapping and elemental analysis of CCM@CDs/CS-NP.

EDX analysis was used to evaluate the quantitative analysis of the synthesized nanoparticles (Figure 3A). The CCM CD-decorated chitosan nanoparticle contains numerous elements, as confirmed by EDX analysis. A majority of the chemical composition of the CCM@CDs/CS-NP is made up of C, O, and N atoms. As a result of EDX analysis, the mass element ratios of the synthesized nanomaterial were determined as 45,27% O, 44,04% C, and 10,69% N. Figure 3B demonstrates the EDX elemental maps of a synthesized novel nanoparticle. The existence of carbon, nitrogen, and oxygen in CCM@CDs/CS-NP is confirmed by EDX elemental mapping derived from the SEM study. In particular, EDX mapping has proven that oxygen and carbon exhibit a regular and dense distribution, whereas nitrogen is observed to be present at a lower concentration due to the chitosan NP-containing amine group.

### 3.3 Analysis of FTIR Spectroscopy

**Figure 4:**
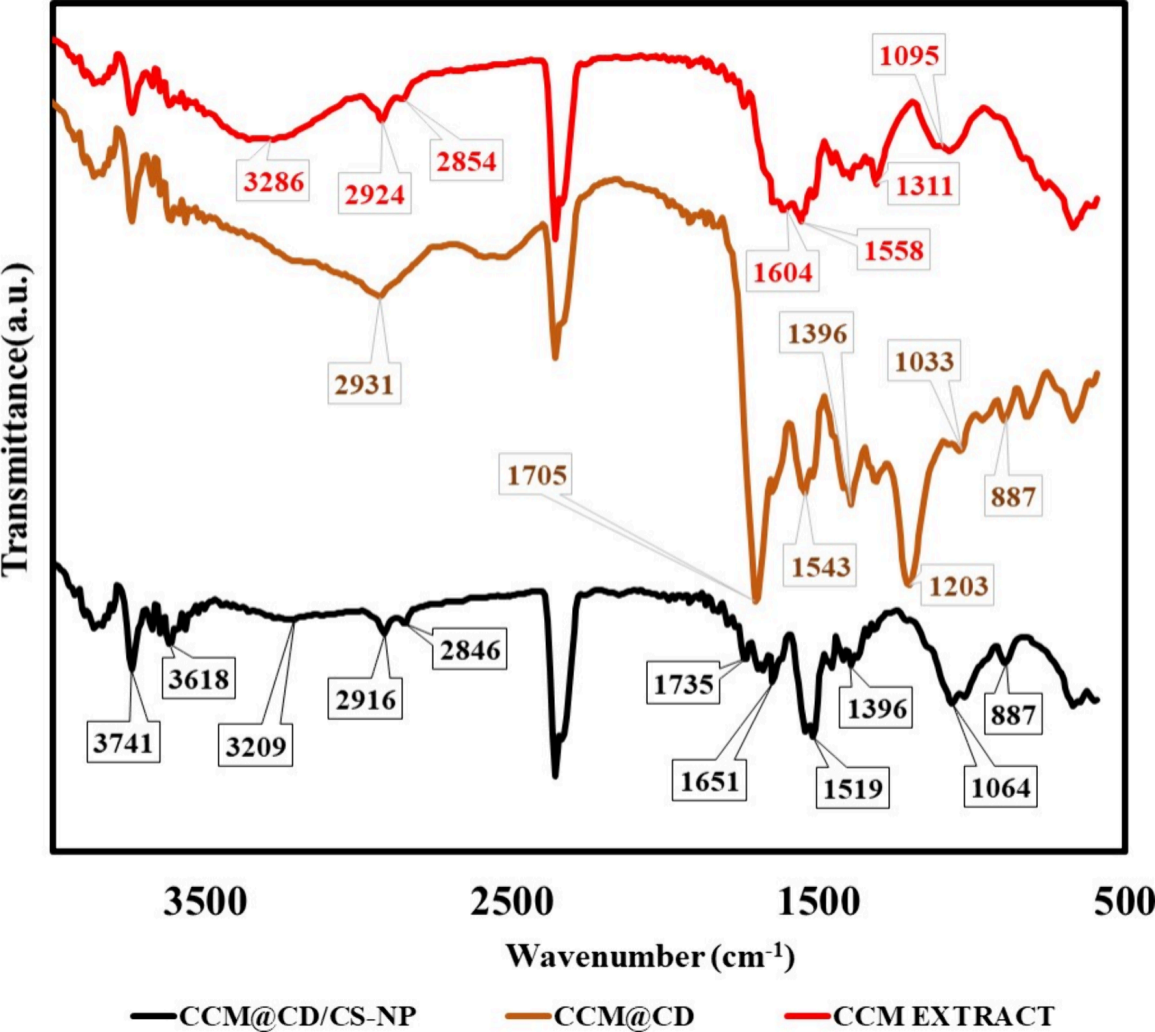
FT-IR spectrum results of curcumin extract, curcumin carbon dot, and curcumin carbon dot-decorated chitosan nanoparticle.

CCM is a chemically purified fraction of turmeric. Curcumin’s extract FTIR spectrum is seen in Figure 4. OH stretching vibrations: wide peak at 3286 cm^–1^ and sharp peak at 3741 cm^–1^ indicate the presence of hydroxyl groups. The peak positions and shapes might suggest the involvement of hydrogen bonding with other functional groups or solvent molecules. v(C=C) and (C=O) vibrations: The prominent peak at 1620 cm^–1^ suggests the presence of double bonds and carbonyl groups. The peak position might shift depending on the electron density around these groups, indicating possible interactions with the neighboring functional groups. C=C ring vibrations: The peak at 1604 cm^–1^ indicates the presence of symmetric aromatic ring stretching vibrations. The peak position can provide insight into the conjugation and delocalization of π-electrons within the aromatic system.

The FT-IR spectrum of CCM@CDs provided OH stretching vibrations: The wide signal at 3421 cm^–1^ indicates the presence of hydroxyl groups. The peak position and shape can reveal information about hydrogen bonding and the surrounding environment. Methyl group C–H vibrations: The bending peak at 2924 cm^–1^ and stretching peak at 1384 cm^–1^ provide information about the nature of the methyl groups and any possible interactions with neighboring functional groups or surfaces.

The spectral analysis of CCM@CDs/CS-NP reveals amine and OH stretching vibrations: The bands at 3209 and 3602 cm^–1^ suggest the presence of the amine and hydroxyl groups. The peak positions and potential shifts might indicate the existence of hydrogen bonding interactions between these groups and other moieties in the composite. C=O vibration: The peak at 1651 cm^–1^ indicates the presence of a carbonyl group that might be involved in hydrogen bonding or other interactions with neighboring functional groups. CH stretching vibration: The peak at 2916 cm^–1^ provides information about the nature of the CH groups and any possible interactions with the surrounding functional groups or surfaces. N–H bending vibration: The peak at 1519 cm^–1^ indicates the presence of an N–H group, which might participate in hydrogen bonding or other interactions within the composite. C–H bending vibration: The peak at 1396 cm^–1^ suggests the presence of a C–H group, which can interact with other functional groups through van der Waals or electrostatic interactions. O–C stretching vibrations: Peaks at 1064 and 1031 cm^–1^ indicate the involvement of O–C stretching vibrations, potentially revealing interactions between these oxygen-containing groups and other components in the composite. These results confirmed the structure of CCM@CDs/CS-NP.

**Figure 5:**
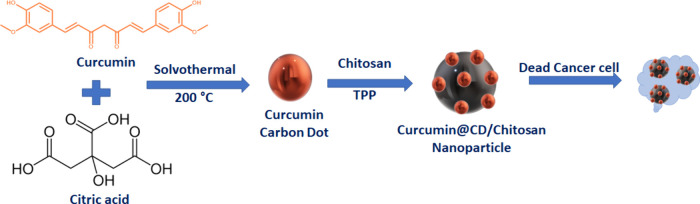
Schematic diagram of the synthesis and anticancer effect of curcumin carbon dot-decorated chitosan nanoparticle.

In this study, a new nanomaterial was synthesized. Water-soluble CCM extract was synthesized as a CCM CD by using citric acid as a carbon source at high temperatures. Then, the obtained CDs were synthesized as chitosan NPs in the presence of TPP with chitosan, which has biocompatible and antimicrobial properties. Thus, the effectiveness of chitosan nanoparticles functionalized with curcumin carbon dots in cancer cell lines was investigated (Figure 5).

### 3.4 Cytotoxic Activity Determination by XTT Assay

The effects of CCM CD-decorated Chitosan NP on cell viability in HepG2 hepatocellular carcinoma cells were measured using an XTT colorimetric cytotoxicity test at 24 and 48 h. Our novel agent had an IC_50_ of 323.61 μg/mL at 24 h and 267.73 μg/mL at 48 h in HepG2 cells. Cell viability was evaluated in terms of both dosage and duration when the effects of adding different concentrations (10–500 μg/mL) to the cells were seen after 24 and 48 h, respectively.

**Figure 6:**
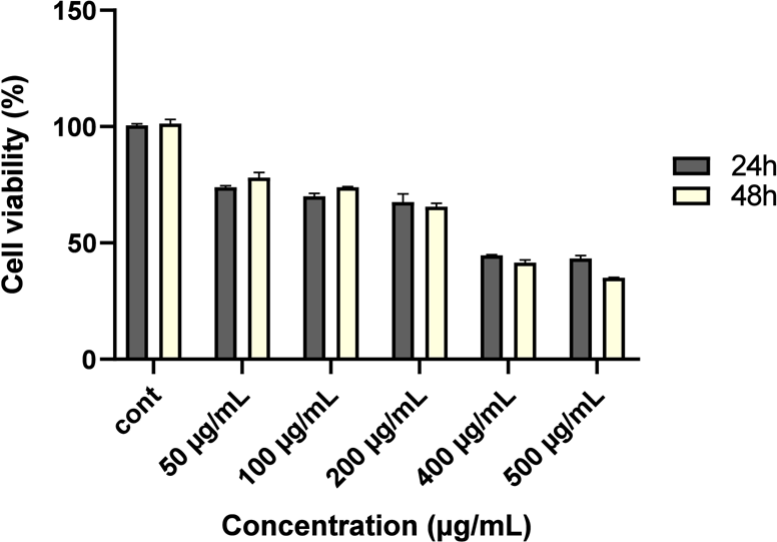
Evaluation of CCM CD-coated chitosan NP with HepG2 hepatocellular carcinoma cells for 24 and 48 h was used to assess the cytotoxic effects of the compound. CCM CD-decorated chitosan NP dosages between 50 and 500 g/mL were used to treat the cells. There were three separate runs of each experiment. Cell growth is inhibited even more through raising the dosage rate. Furthermore, when comparing the effects of the same dosage of medication after 24 and 48 h, it is clear that there are differences. Cell viability is further decreased when the same amount is administered to cells 24 and 48 h later. This demonstrates that the impact of the CCM CD-decorated chitosan NP on HepG2 cells depends on both dosage and exposure duration.

Figure 6 shows the vitality of the HepG2 hepatocellular carcinoma cell line. The extract with the lowest IC_50_ value was tested further in molecular biological tests (real-time-PCR assays) on HepG2 cells.

### 3.5 RT-PCR Results

**Figure 7:**
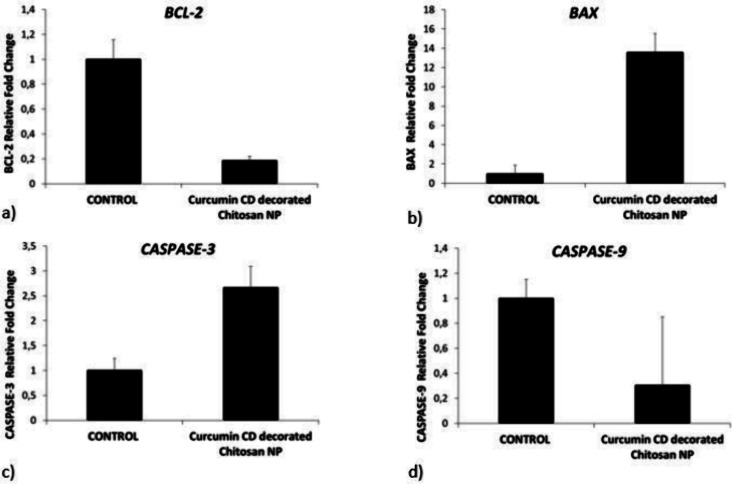
Graphs of *Bax, Bcl-2 Caspase-3,* and *Caspase-9* fold change in HepG2 hepatocellular carcinoma cells compared to control after CCM CD-decorated chitosan NP treatment.

Changes in the mRNA expression of genes involved in the intrinsic process of apoptosis (*Bcl-2, Bax, CASPASE-3,* and *CASPASE-9*) in HepG2 cancer cells were examined by quantitative polymerase chain reaction (qPCR) using SYBR Green Master Mix to determine the effects of CCM CD-decorated chitosan NP. Table 2 and Figure 7 display fold changes and p values for the genes.

**Table 2:** Curcumin CD-Decorated Chitosan NP (267.73 μg/mL at 48 h) Induced Apoptosis in HepG2 Cells as Measured by Changes in mRNA Expression Fold and *p* Values (*p 0.05)

gene names	fold change	*p* value
*BCL-2*	*0.19*	0.006453*
*BAX*	*13.58*	0.002620*
*CASPASE-3*	*2.67*	0.019694*
CASPASE-9	*0.31*	*0.275940*

Compared to the control group, Bcl-2 mRNA expression decreased 5.32 times and Caspase-9 expression 3.28 times in HepG2 hepatocellular carcinoma cells treated with 267.73 μg/mL CCM CD-decorated chitosan NPs after 48 h. Likewise, BAx mRNA expression increased 13.58 times in the dose group cells treated with nanoparticles, while caspase-3 expression increased 2.67 times. When the results obtained were compared statistically, the downregulation of caspase-9 gene expression in the dose group was not found to be significant (*p* > 0.05). However, downregulation of Bcl-2 and upregulation of caspase-3 and BAx gene expressions were found to be statistically significant. All these results show us that nanoparticle treatment may have induced cell death in HepG2 cells through the intrinsic apoptotic mechanism, depending on the activation of caspase-3 mediated by BAX/BCL-2 exchange. Depending on the regulation of apoptotic gene expressions, cell proliferation may be decreased.

## 4 Discussion

The study investigated the potential anticancer effects of CCM CD-decorated chitosan NPs on HepG2 hepatocellular carcinoma cells. The findings demonstrated that the NPs inhibited cancer cell proliferation and triggered apoptosis. In addition, the NPs increased the expression of genes involved in apoptosis and decreased the expression of genes involved in proliferation, indicating that the NPs have the potential to be developed as a medicinal therapy agent for the cure of hepatocellular carcinoma. One of the advantages of using nanoparticles in cancer therapy is their ability to selectively target cancer cells while minimizing toxicity to normal cells. This is due to the NPs’ low dimension, which allows them to penetrate the cell membrane and accumulate in the cancer cells.

Chitosan, a biopolymer composed of chitin, is both biocompatible and biodegradable and occurs naturally in high quantities. Because of its mucoadhesive qualities and potential to produce NPs, it has seen extensive applications as a drug delivery carrier. The use of chitosan nanoparticles in cancer therapy has been extensively studied due to their biocompatibility, biodegradability, and low toxicity.^40^ Boroujeni and groups^41^ created a CCM nanocarrier using folate-modified chitosan. Molecular weight and folate substitution are two further factors that have altered the NP chemistry and physics. The sample with the lowest molecular weight, the highest zeta potential (−6.55), and the maximum loading performance (92.06), Lchitosan/LFA, is ideal for NPs in the range 80–200 nm. In addition, the amine group of chitosan protonates and the resulting polymer structure expand at acidic pH, making CCM release from NPs quicker than at neutral pH. This shows the pH-responsiveness of these NPs. Cell experiments indicated that folate-modified chitosan-loaded NPs may transport curcumin to malignant cells.

**Figure 8:**
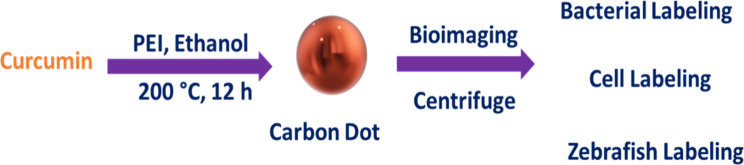
C-dot synthesis via the hydrothermal reactor schematically. Adapted with permission from ref (27). Copyright 2023. ACS.

CCM, a component of turmeric, has been scientifically shown to inhibit tumor growth.^42^ The lack of solubility and bioavailability prevents its widespread use in medicine. CCM’s solubility and bioavailability may be improved by the use of CDs as a carrier. Low toxicity and high biocompatibility make CDs, a kind of fluorescent nanomaterial, a promising new tool in the medical field. Hydrothermal synthesis of a novel surface-passivated CD (CDP) from the environmentally friendly substrate CCM (Figure 8) is described by Pal et al.^27^*E. coli* DH5α and *S. aureus* were utilized as biolabeling microorganisms. Biolabeling and cytotoxicity were very effective in mouse fibroblast (NIH 3T3), lung cancer (A549), and colon cancer (HCT-15) cell lines. Bioimaged zebrafish (ASWT) embryos inferred in vivo toxicity. Synthesized CDs also scavenged free radicals dose-dependently. Unpassivated CDs detected micromolar ferric ions. Our work shows that CDPs may be high-performance optical nanoprobes and biolabeling and contrasting agents. Li and group^43^ developed and synthesized 12 asymmetric and 5 symmetric CCM derivatives. Most compounds had greater antioxidant activity than Vc, and compound 14, which shares substituent groups with CCM, was stronger than CCM. Compound 25 acts like CCM. CCM outperformed all asymmetric and symmetric compounds, notably compound 25, which selectively killed MCF-7 cells. Thus, new asymmetric CCM analogs may be medicinal due to their cytotoxic selectivity and antioxidant properties.

**Figure 9:**
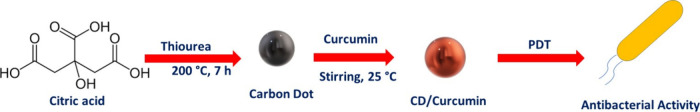
CDs/CCM nanocomposite photosensitizer synthesis. Adapted with permission from ref (44). Copyright 2023. ACS.

Yan and team^44^ improved a compound; nano-photosensitizer (PS)-mediated photodynamic therapy (PDT) has been developed using CDs as carriers for the delivery of CCM, as shown in Figure 9. This system exhibits synergistic photodynamic and photothermal antibacterial effects when it is triggered by dual-wavelength illumination. The combined near-infrared and visible light irradiation leads to the generation of ROS and a moderate temperature increase, effectively damaging bacterial cell membranes. The CDs/CCM nano-PS system is a promising approach for improved antibacterial therapy in photomedicine. Venkatasubbu and group^45^ aimed to enhance wound dressing properties by coating conventional cotton cloth with a CCM nanocomposite. The CCM-coated cotton cloth demonstrated significant improvements, including a 74% increase in the drying time and a 50% boost in water absorbency. Additionally, the CCM nanocomposite exhibited strong antibacterial activity against wound-associated bacterial species. Upadhyayam and colleagues^46^ synthesized water-soluble nanocomposites (NCs) using zinc oxide (*n*-ZnO) and O-carboxymethyl chitosan (O-CMCS) for delivering the anticancer drug CCM. The NCs showed controlled drug release and higher toxicity against cancer cells (MA104) compared to normal cells. These findings suggest the potential of Cr/O-CMCS/n-ZnO NCs as an effective and promising nanomatrix for anticancer therapy and other biomedical applications. ZnO nanoparticles and chitosan-coated ZnO-CCM nanocomposite were synthesized by Deshpande.^47^ The nanocomposite showed good drug encapsulation efficiency, biocompatibility with cells, and potent anticancer activity against melanoma cells. In an in vivo mice model, ZnCurNC effectively inhibited tumor growth compared with untreated mice. The nanocarrier’s aqueous formulation allowed for improved therapeutic efficacy and reduced toxicity associated with organic solvents. However, further toxicological assessments are necessary to ensure the safety of these nanomaterials for skin cancer treatment.

The findings of this study suggest that CCM CD-decorated chitosan nanoparticles have potential as an innovative medicinal therapy compound for the cure of hepatocellular carcinoma. As a crucial step in cancer therapy, the NPs successfully inhibit cancer cell growth and trigger apoptosis. The upregulation of apoptosis-related genes and downregulation of proliferation-related genes further support the anticancer properties of the nanoparticles. However, there are still several challenges that need to be addressed before CCM CD-decorated chitosan NPs can be used as a clinical treatment for the HepG2 cell line. One of the main challenges is optimizing the properties of these nanoparticles, such as their composition of elements, shape, and size, to ensure that they can effectively target HepG2 cells and penetrate the tumor microenvironment. Another challenge is evaluating the efficacy of these nanoparticles in vivo, as many of the studies conducted so far have been in vitro. Further study is required to evaluate the safety and effectiveness of the obtained nanoparticles in animal models and clinical trials.

The study includes some limitations. One of the limitations of the study is that the expression changes of the genes involved in the extrinsic pathway were not investigated or confirmed at the protein level. In addition, the use of healthy normal cell lines is another limitation of the study. The study’s main limitation is that it was conducted in vitro, which means it was performed in a laboratory setting using isolated cells. Further research is required to assess the safety and effectiveness of these NPs in vivo using animal models or clinical trials. Additionally, long-term toxicity studies and investigations into the delivery mechanisms of nanoparticles are necessary before their potential as a therapy can be fully understood.

## 5 Conclusions

HepG2 is a type of liver cancer that resists chemotherapy and radiation. As a result, the development of innovative and effective treatment strategies for HepG2 cell lines is critical. CCM is a naturally occurring molecule that can be found in turmeric. It has been investigated for its possible anticancer effects; however, due to its limited bioavailability, its medicinal potential is restricted. Utilizing nanoparticles to enhance the solubility, stability, and bioavailability of curcumin could solve this problem. The researchers employed chitosan nanoparticles as a carrier for CCM and then coated the surface of the NPs with CDs to increase the amount of CCM that was taken up by cancer cells. According to the findings of the research, the CCM CD-decorated chitosan NPs were successful in preventing the growth of HepG2 cells when tested in vitro. In addition, the NPs caused the HepG2 cells to undergo apoptotic cell death by activating caspase-3 and caspase-9, which are proteins that play a role in controlling the process of cell death. The results of this research show promise for the creation of a new and successful therapy for HCC, using CCM CD-decorated chitosan NPs as a leading candidate. The study was in vitro; therefore, further research is required to assess these NPs’ safety and effectiveness in vivo. The use of nanotechnology in cancer therapy is an emerging topic, and the results of this research show that CCM CD-decorated chitosan NPs have therapeutic promise for HepG2.
